# Intraoperative and early postoperative pain in cats that underwent ovariohysterectomy using a spay hook: a randomised, masked, experimental study

**DOI:** 10.1186/s12917-023-03718-w

**Published:** 2023-09-13

**Authors:** Mareliza Possa de Menezes, Luís Guilherme de Faria, Guilherme Galhardo Franco, Cléber Kazuo Ido, Fernando Yoiti Kitamura Kawamoto, João Augusto Leonel de Souza, Paula Regina Silva Gomide, Fabrícia Geovânia Fernandes Filgueira, Diego Iwao Yamada, Bruno Watanabe Minto

**Affiliations:** 1 Department of Clinic and Veterinary Surgery, School of Agricultural and Veterinarian Sciences, São Paulo State University (UNESP), Via de Acesso Prof. Paulo Donato Castellane w/n, Jaboticabal - São Paulo, CEP 14884-900 Brazil; 2 University of Uberaba (UNIUBE), Uberaba – Minas Gerais, 38.055-500 Brazil; 3 Center of Agrarian Sciences and Engineering, Federal University of Espírito Santo (UFES), Alegre - Espírito Santo, 29.500-000 Brazil; 4 University Center of Lavras (UNILAVRAS), Lavras – Minas Gerais, 37203-593 Brazil; 5 University of Araraquara (UNIARA), Araraquara, São Paulo, 14801-340 Brazil; 6 University of Marília (UNIMAR), Marília, São Paulo, 17525–902 Brazil

**Keywords:** Feline, Hook technique, Surgical sterilization, Surgical time

## Abstract

**Background:**

This study aimed to compare the feasibility and practicality of the ovariohysterectomy (OHE) technique in cats with or without a spay hook with respect to the incision size, surgical time, surgical variables, and intra- and postoperative pain. Twenty-nine female cats underwent OHE using a spay hook (spay hook group [SHG], n = 15) or without using a spay hook (control group [CG], n = 14) to achieve the ovaries and cervix. Physiological parameters were monitored during the intraoperative period, and postoperative pain was assessed using a multidimensional composite and visual analogue pain scales.

**Results:**

The SHG had a significantly shorter operative time than the CG. The variables in the intraoperative period showed no statistically significant difference between both groups, as well as the early postoperative pain.

**Conclusions:**

Less invasive OHE using a spay hook could potentially be a viable and feasible technique when performed by an inexperienced surgeon with appropriate training, especially in sterilisation campaigns, reducing the time to perform the procedure and increasing the number of animals spayed per time.

## Background

Ovariohysterectomy (OHE) is the surgical removal of the ovaries and uterus [1] and has been reported as the most common elective surgery in small animal practice [2]. This surgery can be used as a treatment for female genital diseases in dogs and cats, such as pyometra or uterine/ovarian neoplasia [1, 3, 4] and feline mammary fibroepithelial hyperplasia [5]. However, elective surgical sterilisation, aimed at population control or prevention of diseases associated with the reproductive system, is the most common indication for OHE in dogs and cats [1, 3].

Several techniques, including open and laparoscopic approaches, have been described for performing OHE [3, 6–10]. In previous studies, laparoscopic surgery showed some advantages over laparotomy, such as better lighting and magnification of the organs approached during the procedure, reduction of pain and infection rate in the postoperative period, and faster recovery [6, 11–19]. However, this technique also has several limitations. Laparoscopic procedures require specialised surgical equipment and a well-trained team with a longer learning curve [11]. These requirements could increase the cost of equipment and human resources, which is an important concern in low- and middle-income countries [19]. In these countries, large-scale surgical sterilisation is often required to control the population of dogs and cats [20].

As an alternative, a more minimally invasive, non-laparoscopic surgical approach could contribute to reducing pain, complication rates, recovery time, and costs. These minimally invasive surgical techniques are characterised by a reduction in the length of the anatomic approach without sacrificing precision and efficiency [21], causing less tissue damage to the abdominal wall without using laparoscopic devices [22, 23]. Thus, the use of a spay hook for exteriorising the uterus can be a low-cost alternative and has been frequently used by veterinarians [24].

Several studies have already reported different techniques to spay cats [1, 3, 7, 25–28], as well as the differences in the complication rates and postoperative pain between OHE and ovariectomy (OVE) [28, 29] and between laparoscopic, laparoscopic-assisted, and conventional techniques [19, 30]. However, to date, no study has compared intraoperative and early postoperative pain between the OHE approaches with and without the use of a spay hook. The spay hook in an OHE procedure could be useful in low- and middle-income settings to guarantee good practices and animal welfare at low cost.

In this sense, this randomised, masked, experimental study aimed to compare intraoperative physiological measures and pain evaluation in the intraoperative and early postoperative period in cats undergoing OHE with and without the use of a spay hook, both by celiotomy approaches.

## Results

Thirty-three cats were initially selected; however, four were excluded because of aggressive behaviour (n = 1), ascites (n = 1), and physiological parameter outliers (n = 2).

The body weight (mean ± standard deviation [SD], CG, 2.87 ± 0.44 kg; SHG, 2.71 ± 0.54 kg) was not different between the groups.

The surgical time and incision size significantly differed between the groups. The technique in the control group (CG) was longer than in the spay hook group (SHG) (21.07 ± 4.73 and 15.71 ± 2.27 min, respectively; *p* < 0.05; Table 1) with a longer length incision (5.29 ± 1.14 and 1.97 ± 0.57 cm, respectively; *p* < 0.001; Table 1). The anaesthesia time was not different between the groups (*p* > 0.05, Table 1).

**Table 1:** Descriptive analysis to compare weight, length of the incision, and anaesthetic and operative time

Variable	Group	n	Mean ± SD	Minimum	Maximum	*p-*value
Weight (Kg)	CG	13	2.87 ± 0.44	2.0	3.5	0.4058
	SHG	15	2.71 ± 0.54	1.8	3.8	
Lenght of incision (cm)	CG	14	5.29 ± 1.14	2.6	7.5	1.32 × 10^− 10^
	SHG	15	1.97 ± 0.57	1.0	3.0	
Anesthesic time (min)	CG	14	31.21 ± 9.46	20.0	55.0	0.0912
	SHG	13	25.62 ± 6.76	15.0	39.0	
Operative time (min)	CG	14	21.07 ± 4.73	14.0	32.0	0.0007
	SHG	14	15.71 ± 2.27	13.0	21.0	

CG: Control groups; SHG: Snook Hook Group; n: number of observations; SD: standard deviation. Values significantly different: *p* < 0.05.

### Intraoperative measures

Baseline heart rate (HR) and respiratory rate (RR) were similar between the groups. The RR did not differ between the groups or among the time points observed from the time point immediately before the anaesthesia induction (IND). The HR showed differences between the groups at all time points. The systolic blood pressure (SBP) was significantly higher in both groups at the time point during the first pedicle clamping (P2) and time point during the second pedicle clamping (P3) (*p* < 0.05). The rectal temperature (RT) was significantly higher in both groups at the IND and time point immediately after the anaesthesia induction and before the fentanyl administration (P0) and lower at the time point at the end of the skin closure (P6). Oxyhaemoglobin saturation (SPO_2_) only significantly differed between the time point during the uterus clamping (P4) and P6 in both groups (Table 2).

**Table 2:** Physiological parameters of cats in the CG and SHG during the intraoperative period

Variable	Group	Baseline	PAM	IND	P0	P1	P2	P3	P4	P5	P6
ETiso	CG	-	-	1.03 ± 0.05^a^	1.11 ± 0.05^b^	1.09 ± 0.05^ab^	1.09 ± 0.05^ab^	1.13 ± 0.05^b^	1.10 ± 0.05^ab^	1.13 ± 0.05^b^	1.15 ± 0.05^b^
	SHG	-	-	1.05 ± 0.05^a^	1.13 ± 0.05^b^	1.11 ± 0.05^ab^	1.11 ± 0.05^ab^	1.15 ± 0.05^b^	1.13 ± 0.05^ab^	1.15 ± 0.05^b^	1.17 ± 0.05^b^
ETCO2	CG	-	-	25.07 ± 1.35^a^	25.46 ± 1.33^a^	25.14 ± 1.34^a^	29.63 ± 1.33^c^	29.12 ± 1.33^bc^	28.84 ± 1.33^bc^	27.23 ± 1.33^abc^	26.91 ± 1.33^ab^
	SHG	-	-	25.07 ± 1.35^a^	25.46 ± 1.33^a^	25.14 ± 1.34^a^	29.63 ± 1.33^c^	29.12 ± 1.33^bc^	28.84 ± 1.33^bc^	27.23 ± 1.33^abc^	26.91 ± 1.33^ab^
SBP (mmHg)	CG	-	-	82.25 ± 4.59^a^	81.31 ± 4.56^a^	88.23 ± 4.56^ab^	106.39 ± 4.55^d^	106.1 ± 4.57^d^	103.28 ± 4.56^ cd^	96.15 ± 4.55^bc^	93.09 ± 4.56^b^
	SHG	-	-	75.63 ± 4.46^a^	74.69 ± 4.45^a^	81.62 ± 4.45^ab^	99.77 ± 4.42^d^	99.48 ± 4.44^d^	96.66 ± 4.44^ cd^	89.53 ± 4.42^bc^	86.47 ± 4.45^b^
RT (ºC)	CG	-	-	37.55 ± 0.2^e^	37.39 ± 0.2^de^	37.26 ± 0.2^d^	36.96 ± 0.2^c^	36.73 ± 0.2^c^	36.49 ± 0.2^b^	36.28 ± 0.2^b^	35.95 ± 0.2^a^
	SHG	-	-	37.35 ± 0.2^e^	37.19 ± 0.2^de^	37.06 ± 0.2^d^	36.75 ± 0.2^c^	36.52 ± 0.2^c^	36.28 ± 0.2^b^	36.08 ± 0.2^b^	35.74 ± 0.2^a^
SPO2 (%)	CG	-	-	97.9 ± 0.31^ab^	97.87 ± 0.31^ab^	97.93 ± 0.31^ab^	98.36 ± 0.31^ab^	98.47 ± 0.31^b^	98.1 ± 0.31^ab^	97.87 ± 0.31^ab^	97.8 ± 0.31^a^
	SHG	-	-	98.3 ± 0.3^ab^	98.26 ± 0.3^ab^	98.32 ± 0.3^ab^	98.75 ± 0.3^ab^	98.86 ± 0.3^b^	98.49 ± 0.3^ab^	98.26 ± 0.3^ab^	98.19 ± 0.3^a^
HR (bpm)	CG*	185.55 ± 6.31^c^	187.48 ± 6.56^c^	161.09 ± 6.32^ab^	157.15 ± 6.26^ab^	148.15 ± 6.26^a^	181.88 ± 6.26^c^	187.53 ± 6.26^c^	182.08 ± 6.26^c^	174.53 ± 6.26^bc^	160.32 ± 6.26^ab^
	SHG*	163.47 ± 6.33^c^	165.4 ± 6.56^c^	139.01 ± 6.24^ab^	135.07 ± 6.2^ab^	126.07 ± 6.2^a^	159.79 ± 6.2^c^	165.45 ± 6.2^c^	160 ± 6.2^c^	152.45 ± 6.2^bc^	138.24 ± 6.2^ab^
RR (mpm)	CG	49.75 ± 2.15^b^	50.81 ± 2.31^b^	28.04 ± 2.18^a^	24.87 ± 2.15^a^	21.83 ± 2.15^a^	25.9 ± 2.15^a^	24.52 ± 2.15^a^	25.21 ± 2.15^a^	23.42 ± 2.15^a^	21.42 ± 2.15^a^
	SHG	46.31 ± 2.17^b^	47.36 ± 2.28^b^	24.6 ± 2.14^a^	21.42 ± 2.13^a^	18.39 ± 2.13^a^	22.46 ± 2.13^a^	21.08 ± 2.13^a^	21.77 ± 2.13^a^	19.97 ± 2.13^a^	17.97 ± 2.13^a^

Data are expressed as mean ± SD. HR Heart rate expressed in beats per minute; RR Respiratory rate expressed in movements per minute; RT Rectal temperature in Celsius; SBP Systemic Blood Pressure in mmHg; CG Control Group; SHG Snook Hook Groups; IND immediately before the anesthesia induction; P0 immediately after the anesthesia induction and before the fentanyl administration, P1 *linea alba* incision, P2 first pedicle clamping; P3 second pedicle clamping, P4 uterus clamping, P5 abdominal wall closure, P6 at the end of the skin closure.*Value significantly different (*p* < 0.05) between groups. Letters means values significantly different among the groups in each time point, as b > a.

### Pain assessment

**Fig. 1:**
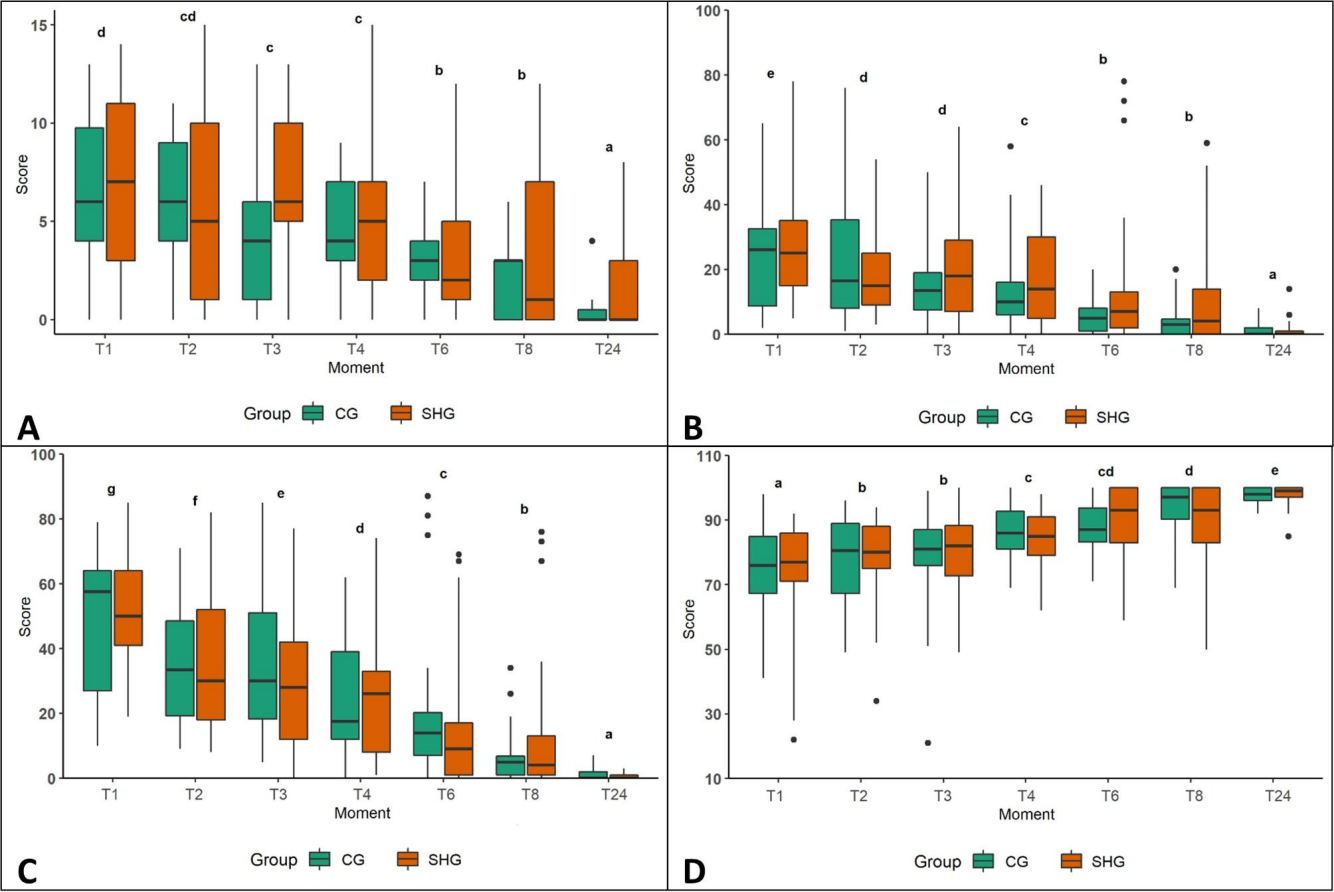
Box plot showing median, interquartile range, minimum and maximum pain, analgesia, and sedation scores. Legend: CG: Control Group; SHG: Snook Hook Group. Multidimensional composite pain scale (A); visual analogue scale (VAS) for pain (B); VAS for sedation (C); VAS for analgesia (D)

Pain, sedation, and analgesia scores did not significantly differ (*p* > 0.05) between the groups. The sedation scores decreased in both groups from T1 (1 h after the end of the procedure) to T24 (24 h after the end of the procedure), and the analgesia scores increased in both groups from T1 to T24. Pain scores were higher at T1 in both groups (Fig. 1).

The prevalence of rescue analgesia at some time point during the observed 24 h was lower in the CG (7/14, 50%) than in the SHG (11/15, 73.3%). However, no significant difference was found in the number of rescue analgesia needed between the groups (*p* = 0.25; CG, 0.5, minimum of 0 and maximum of 3; SGH, 1, minimum of 0 and maximum of 6) or in the prevalence (*p* = 0.36). Rescue analgesia was administered at T1 (CG, 5/14; SHG, 6/15), T2 (CG, 5/14; SHG, 4/15), T3 (CG, 2/14; SHG, 7/15), T4 (CG, 2/14; SHG, 4/15), T6 (CG, 0/14; SHG, 2/15), T8 (CG, 0/14; SHG, 3/15), and T24 (CG, 0/14; SHG, 1/15).

### Perioperative complications

No perioperative complications were observed in either group over the observed period.

## Discussion

In this study, we compared perioperative pain and associated complications between two different non-laparoscopic OHE surgical techniques to spay healthy female cats. This study attempted to reproduce low- and middle-income clinical settings, where elective sterilisation is commonly performed during mass sterilisation campaigns.

No significant differences were observed between the CG and SHG regarding the intraoperative physiological parameters and early postoperative pain, except for the HR, which was higher in the CG. However, the HR alone is not necessarily indicative of pain in cats [31–34]. These findings suggest that the use of the spay hook did not cause more tissue trauma during the surgery, which is a safe way to achieve the ovaries and uterus, partly corroborating the findings described by Minto et al. (2021) in a similar study performed in dogs [35].

The RT decreased over time. This finding was expected because of central nervous system depression caused by isoflurane inhalation. The sensitivity of the thermoregulatory centre of the hypothalamus is decreased by this anaesthetic. Another possible explanation for the decrease in RT may be related to the size of the animal (< 5 kg), which, because of its larger surface area-to-volume ratio, has a higher risk of developing hypothermia [36]. Furthermore, acepromazine maleate could contribute to hypothermia owing to its vasodilation properties [37]. Pereira et al. also reported a decrease in RT in female cats that underwent OHE and OVE [29].

The end-tidal carbon dioxide (ETCO_2_), the oxyhemoglobin saturation (SPO_2_), HR, and SBP were higher at P2, P3, and P4 in both groups. These findings could be explained by the fact that during these time points, the ovarian pedicles were traction, clamped, ligated, and transacted, providing greater nociception stimulation [38]. Furthermore, this corroborates that the size of the musculature and skin incisions is likely not an important factor in increasing intraoperative pain.

The postoperative pain scores (multidimensional composite scale [MCS] and visual analogue scale [VAS]) decreased over time (from T1 to T24) in both groups, which is expected in cats undergoing OHE [38]; however, it was not significantly different between the groups. Previous studies performed on bitches and cats undergoing OHE or OVE have reported similar findings [39, 40]. At the same time, the VAS analgesia score increased, and the VAS sedation scores decreased along the time points, which means that the analgesic protocol effectively managed pain and improved analgesia without increasing sedation. As the anaesthetic protocol and pain management were the same for both groups, we can conclude that early postoperative pain did not differ between the techniques.

Even though the number of rescue analgesia performed in the SHG was higher than that in the CG, no statistically significant difference was observed between the groups (*p* > 0.05). We supposed that this was probably due to individual variations in response to the analgesic drugs, because only one cat in the SHG group reached a score above the cutoff values in the scales.

It is difficult to reliably measure blood pressure in non-anaesthetised or sedated cats is difficult in a reliable way [29, 41, 42]. Therefore, in this study, the SBP was not evaluated in the postoperative period. This fact did not interfere with our results because this MCS has already been validated for use considering each of the subscales and not the full version. According to the Receiver operating characteristic (ROC) curve analysis, rescue analgesia was strongly recommended in cats, reaching at least 33.3% of the total points in the MCS [43].

The only statistically significant differences between the groups were the incision length and duration of surgery, which were shorter in the SHG (*p* < 0.001). This finding differs from that reported by Minto et al. (2021) in dogs, in which they did not find a significant difference in the operative time [35]. In this study, the mean operative times were 15.71 ± 2.27 and 21.07 ± 4,73 min in the SHG and CG, respectively. Both were shorter than the operative time reported in previous studies with laparoscopic and laparoscopic-assisted techniques of OHE or OVE in cats [19, 44, 45]. They were similar to the time in non-laparoscopic OHE previously reported, ranging from 12 to 14 min [19, 37, 46, 47], and considerably longer than the time reported by Miller et al. (2016) [28]. These differences observed in the operative times in different studies could be explained by the surgeon’s experience in performing OHE.

The anaesthetic time was shorter in the SHG; however, we did not find any statistically significant difference in the mean anaesthetic times between the groups. This could be explained by the fact that the anaesthetic protocol was the same for both groups, and the differences in the operative and anaesthetic times between the groups were similar. Once the time increases and the difference remains the same, it is expected that there will be no statistically significant difference when we compare the anaesthetic times. So, we can conclude that the difference found in this study is only regard to techniques used. No major complications were observed in either technique, similar to previous reports [19, 37, 44, 46, 47].

A prolonged surgery time is one of the main causes of surgical site infection, which could contribute to suture dehiscence and increase in morbidity and the duration of hospitalisation and recovery [6, 14, 15]. According to the literature, the surgical site infection rate double each hour during the procedure [48]. However, we could not conclude that the shorter surgical time in the SHG would contribute to a decrease in the surgical site infection rate because we did not evaluate the wound after 24 h postoperatively. Therefore, further studies are warranted.

The use of contraceptive drugs is associated with the development of reproductive/mammary tumours. Moreover, safe immunocontraceptives and drugs for chemical castration are under development. Surgical sterilisation has been the main method of dog population control [49–52]. However, this method is necessary in low- and middle-income countries, where free-roaming dogs are a concern [49]. In these clinical settings, many cats and dogs must be spayed within a short period with minimal costs. Therefore, a significant reduction in surgical time could be beneficial.

This study compared two simple surgical techniques that can be performed in any clinical setting. In this study, OHE using a spay hook appeared to be a safe, viable, and feasible technique if performed by a trained and experienced surgeon, especially during sterilisation campaigns. These findings corroborated those of a previous study performed in bittches [35]. Reducing the surgical time and the cost related to the procedure could contribute to increasing the number of animals neutering and preserving animal welfare at a low cost, providing a high-quality, high-volume spay, and neutrality. However, the spay hook technique when performed by a non-trained surgeon or when adhesions to the visceral peritoneum are present could cause iatrogenic injury and increase morbidity [53].

One of the limitations of the present study was that complications from 24 h were not evaluated. Further studies are necessary to compare these two techniques from the 24-h postoperative period, especially regarding surgical site infection rates.

## Conclusions

No clinical or statistically significant differences in intraoperative physiological measures, early postoperative pain, or intraoperative and early postoperative complications were observed in this study between two OHE techniques, with and without the use of a spay hook. OHE using a spay hook showed a significant reduction in operative time and incision length. Less invasive OHE using a spay hook could potentially be a viable and feasible technique when performed by an inexperienced surgeon with appropriate training. This could be beneficial, especially in sterilisation campaigns, by reducing the time to perform the procedure and increasing the number of animals spayed per time.

## Methods

### Ethical aspects

This study followed the recommendations of the Brazilian National Council for the Control of Animal Experimentation (“Conselho Nacional de Controle e Experimentação Animal” - CONCEA) and was approved by the Institutional Ethics Committee on the Use of Animals (“Comissão de Ética no Uso de Animais” – CEUA; protocol number 018336/14). Written informed consent was obtained from the caregivers (shelter) before inclusion into the study.

### Study design and animals

This randomised, masked, experimental study was performed at the Veterinary Hospital of the School of Agrarian and Veterinary Sciences, São Paulo State University, Jaboticabal, São Paulo, Brazil. Twenty-nine healthy intact female domestic cats of mixed breed, that would undergo surgical sterilisation at the Feline Contraception Campaign, promoted by the “Associação Protetora dos Animais” (APA-Animal Protect Association), Jaboticabal, Sao Paulo, Brazil, were selected for this study. All animals were deemed healthy (ASA status I) based on their medical history, physical examination, and laboratory tests (complete blood count and serum chemistry profile).

The exclusion criteria included the following: animals weighting < 1.5 and > 3.5 kg, animals aged < 1 or > 8 years, laboratory test alterations, aggressive behaviour, history of excessive fear on handling, oestrus, pregnancy, lactation, gross reproductive abnormalities, signs of pre-existing pain or inflammation, and underlying diseases.

### Experimental groups

A number (1–33) was assigned to each cat immediately before the procedure. The cats were randomised into one block with an allocation ratio of 1:1. An individual not involved in pain assessment performed the randomisation using a randomisation plan generator (www.randomization.com), allocating each cat to one of the two treatment groups, namely, the CG (n = 14) and SHG (n = 15). Animals in the CG were subjected to OHE without using the spay hook, and animals allocated to the SHG were subjected to OHE using a spay hook to achieve the ovaries and uterus.

### Preoperative preparation and ambience

Forty-eight hours prior to the beginning of the study, the cats were clinically examined and acclimatised to the observers, cages, and environment. All animals were housed in a restricted circulation room at the Veterinary Hospital outside clinical practice. Food was withheld 12 h prior (overnight fasting), and water was withheld 4 h prior.

### Anaesthetic procedure

A single anaesthesiologist performed all procedures. As premedication, all cats received morphine (0.2 mg/kg) and acepromazine maleate (0.05 mg/kg) intramuscularly. After 15–30 min, a 22- or 24-gauge catheter was placed in the cephalic vein for fluid (Lactated Ringer’s solution) and drug administration. Anaesthesia was induced using propofol (3–5 mg/kg). Orotracheal intubation was performed, and anaesthesia was maintained with isoflurane diluted in 100% oxygen in a circular anaesthesia circuit. Immediately before surgical incision, fentanyl (0.0025 mg/kg) was administered intravenously.

The HR, RR, sPO_2_, RT, and ventilatory parameters were monitored using a multiparameter bedside monitor. The ETCO_2_ and end-tidal isoflurane concentrations (ET_iso_) were measured using a gas analyser. Noninvasive SBP was measured using the ultrasonic Doppler method.

At the end of the procedure, cats in both groups received meloxicam (0.1 mg/kg) and sodic dipyrone (25 mg/kg) intravenously at skin closure and subcutaneously 24 h after hospital discharge. Tramadol hydrochloride (2 mg/kg) was subcutaneously administered 8 h after the end of the procedure in all animals that did not receive rescue analgesia and for all animals 24 h after the end of the procedure. Rescue analgesia was administered intramuscularly using morphine (0.2 mg/kg). Meloxicam (0.1 mg/kg, PO, q. 24 h for 1 day), sodic dipyrone (25 mg/kg, PO, q. 24 h for 3 days), and tramadol hydrochloride (2 mg/kg, PO, q. 12 h for 3 days) were prescribed at discharge for all cats.

### Surgical Procedures

The same two surgeons, one main surgeon with > 3 years of experience and one assistant surgeon, performed all surgeries. Hand antisepsis was performed with surgical scrubbing of 2% chlorhexidine gluconate or 2% povidone-iodine. Hair was clipped at the surgical site, and skin antisepsis was performed with a 2% chlorhexidine scrub and 70% isopropyl alcohol.

After preparation of a wide sterile surgical field according to standardised surgical protocols, ventral midline celiotomy was performed. In the CG, an incision was made from the caudal border of the umbilicus to the cranial third of the caudal abdomen. In the SHG, the incision started from the middle third of the caudal abdomen to the cranial third of the caudal abdomen. Skin and subcutaneous tissue incisions were made with a scalpel, and bleeding was controlled with haemostatic clamps. The *linea alba* incision was the same length as the skin incision. The uterine horns were located, traced cranially, and the ovaries were exteriorized, one by one, using a spay hook (SHG) or surgeon’s fingers (CG). A window was created on the broad ligament to facilitate the isolation of the ovarian pedicle. The ovarian pedicle was clamped with three haemostatic forceps, and a double encircling ligature with 2–0 poliglecaprone 25 was placed proximal to (below) the ovarian pedicle clamps or forceps. Transaction of the ovarian pedicles was performed distal to the haemostatic forceps placed cranial to the planned transection site. Bilateral defects were created in the mesometrium at the level of the caudal uterine body, and broad ligaments were dissected. One encircling ligature and one transfixing-encircling ligature with 2 − 0 poliglecaprone 25 were placed 0.5–1.0 cm caudal to the uterine bifurcation before the uterine transection. The ovaries and uterus were removed, and the uterine pedicle was repositioned to its normal position. All ligatures were inspected for bleeding. The abdominal wall, subcutaneous tissue, and skin closure were performed with 2 − 0 poliglecaprone 25, 3–0 poliglecaprone 25, and 3 − 0 polyamide, respectively, all with a simple interrupted pattern.

### Intraoperative measures

The size of the incision, duration of the procedure (the time from the beginning of the skin incision to its complete closure), and duration of anaesthesia (the time from induction to extubation) were recorded. The following variables were measured during the intraoperative period: HR, RR, SBP, RT, sPO_2_, ETCO_2_, and ETiso. All parameters were recorded at the following time points: immediately before anaesthesia induction (IND), immediately after anaesthesia induction and before fentanyl administration (P0), during the *linea alba* incision (P1), during the first pedicle clamping (P2), during the second pedicle clamping (P3), during uterus clamping (P4), during abdominal wall closure (P5), and at the end of skin closure (P6).

### Pain assessment

During the intraoperative period, pain was assessed based on changes in physiological parameters at IND, P0, P1, P2, P3, P4, P5, and P6. Postoperative pain was subjectively assessed using an MCS, as described for the feline species [29–31], and a VAS. Three masked observers (one master’s student and two PhD students) were trained on www.animalpain.com.br. Postoperative pain was assessed at the following time points after the end of the procedure: T1 (1 h), T2 (2 h), T3 (3 h) T4 (4 h), T8 (8 h), and T24 (24 h). The SBP was not considered in the MCS pain assessment (subscale 3, physiological variables) as most awake cats appeared stressed during the measurement. Thus, the maximum MCS score was 27.

Sedation was assessed using the VAS at the same time points. Rescue analgesia was administered if the cat scored greater than 7 on the MCS or 4 on the VAS.

### Perioperative complications

Adverse effects, such as intra-abdominal haemorrhage, erythema, swelling, vaginal discharge, urinary incontinence, suture dehiscence, and signs of infections, were observed for the first 24 h after surgery.

### Statistical analysis

Statistical analyses were performed using R Core Team (2020)® software. Statistical significance was defined as *p* < 0.05 (two-tailed). Results are presented as mean ± SD for normally distributed continuous variables or median and quartile interval for ordinal or non-normally distributed variables.

To measure the sample size, a power analysis (Fisher’s exact test) was performed, which indicated a sample size of at least 12 animals per group to detect a difference of 80%, with an α level of 5%.

Weight, size of the incision, anaesthesia time, and surgical time were compared between the groups using unpaired t-tests. In all analyses with parametric variables, the residues were normal according to the Shapiro–Wilk test, and homoscedasticity was assessed using the Levene test. Values with standardised residues higher than three times the quartile interval were outliers and were removed from the analysis. For the variable number of analgesia rescue, the group effect was analysed using the non-parametric Mann–Whitney test. The linear mixed model with animal as a random effect compared the groups and time points for the intraoperative parameters. Generalised linear mixed model (non-parametric analysis) with animals and evaluators as a random effect compared the postoperative parameters. In both cases, contrasts between the factors were obtained using the Bonferroni test. The chi-square test was used to compare the prevalence of rescue analgesia.

## Data Availability

The datasets used and/or analysed during the current study are available from the corresponding author upon reasonable request.

## Abbreviations

CG: Control group
ETCO_2_: End-tidal carbon dioxide
ET_iso_: End-tidal isoflurane concentration
HR: Heart rate
IND: Time point immediately before the anaesthesia induction
MCS: Multidimensional composite scale
OHE: Ovariohysterectomy
OVE: Ovariectomy
P0: Time point immediately after the anaesthesia induction and before the fentanyl administration
P1: Time point during the *linea alba* incision
P2: Time point during the first pedicle clamping
P3: Time point during the second pedicle clamping
P4: Time point during the uterus clamping
P5: Time point during the abdominal wall closure
P6: Time point at end of the skin closure
RR: Respiratory rate
RT: Rectal temperature
SBP: Systolic blood pressure
SD: Standard deviation
SHG: Spay hook group
sPO2: Oxyhemoglobin saturation
VAS: Visual analogue scale
